# Threshold tracking transcranial magnetic stimulation and neurofilament light chain as diagnostic aids in ALS

**DOI:** 10.1002/acn3.52095

**Published:** 2024-06-19

**Authors:** Anna B. Jacobsen, Hugh Bostock, James Howells, Bülent Cengiz, Gintaute Samusyte, Martin Koltzenburg, Hossein Pia, Anders Fuglsang‐Frederiksen, Jakob Blicher, Izabella Obál, Henning Andersen, Hatice Tankisi

**Affiliations:** ^1^ Department of Clinical Neurophysiology Aarhus University Hospital Aarhus N 8200 Denmark; ^2^ UCL Queen Square Institute of Neurology Queen Square London WC1N 3BG UK; ^3^ Central Clinical School, Faculty of Medicine and Health University of Sydney Sydney NSW 2006 Australia; ^4^ Department of Neurology Gazi University Faculty of Medicine Beşevler 06570 Ankara Turkey; ^5^ Department of Neurology, Medical Academy Lithuanian University of Health Sciences Kaunas 44307 Lithuania; ^6^ Department of Neurology Lithuanian University of Health Sciences Hospital Kauno Klinikos Kaunas 50161 Lithuania; ^7^ Department of Clinical Neurophysiology National Hospital for Neurology and Neurosurgery Queen Square London WC1N 3BG UK; ^8^ Department of Neurology Aalborg University Hospital Aalborg 9000 Denmark; ^9^ Department of Neurology Aarhus University Hospital Aarhus N 8200 Denmark

## Abstract

**Objective:**

There is a need for sensitive biomarkers in amyotrophic lateral sclerosis (ALS), to enable earlier diagnosis and to help assess potential treatments. The main objective of this study was to compare two potential biomarkers, threshold‐tracking short‐interval cortical inhibition (T‐SICI), which has shown promise as a diagnostic aid, and neurofilament light chains (NfL).

**Methods:**

Ninety‐seven patients with ALS (mean age 67.1 ± 11.5 years) and 53 ALS mimics (aged 62.4 ± 12.9) were included. Mean disease duration was 14 months ±14.1. Patients were evaluated with revised ALS functional rating score (ALSFRS‐R), Penn upper motor neuron score (UMNS), muscle strength using the Medical Research Council (MRC) score and examined with T‐SICI, quantitative electromyography (EMG), and NfL measured in spinal fluid.

**Results:**

NfL increased with increasing UMNS (*rho* = 0.45, *p* = 8.2 × 10^−6^) whereas T‐SICI at 2.5 ms paradoxically increased toward normal values (*rho* = 0.53, *p* = 1.9 × 10^−7^). However, these two measures were uncorrelated. Discrimination between ALS patients and mimics was best for NfL (area under ROC curve 0.842, sensitivity 84.9%, specificity 83.5%), compared with T‐SICI (0.675, 39.6%, 91.8%). For the patients with no UMN signs, NfL also discriminated best (0.884, 89.3%, 82.6%), compared with T‐SICI (0.811, 71.4%, 82.6%). However, when combining NfL and T‐SICI, higher AUCs of 0.854 and 0.922 and specificities of 93.8 and 100 were found when considering all patients and patients with no UMN signs, respectively.

**Interpretation:**

Both T‐SICI and NfL correlated with UMN involvement and combined, they provided a strong discrimination between ALS patients and ALS mimics.

## Introduction

Amyotrophic lateral sclerosis (ALS) is a heterogeneous and complex neurodegenerative disease characterized by a progressive loss of motor neurons leading to a fatal outcome within an average of 3 years after diagnosis of the disease.^1^, ^2^ One of the many challenges in finding a cure for the disease is the lack of sensitive biomarkers to enable earlier diagnosis and to help assess treatment trials. The recent implementation of the Gold Coast Criteria has simplified the criteria for establishing the diagnosis and has increased the diagnostic sensitivity.^3^, ^4^ However, a significant diagnostic delay from symptom onset still remains, especially in patients that in a clinical context are difficult to differentiate from ALS‐mimicking disorders.^5^

An expanding number of studies have evaluated potential biomarkers, and one of the most promising in recent years is the neurofilament light chain (NfL). NfL is a predictor of damage and degeneration of large myelinated axons.^6^ Elevated levels are considered a reliable marker of acute and chronic neuronal injury among other neurodegenerative disorders such as ALS, where it is applied in many clinics to support the diagnosis.^7^, ^8^, ^9^ In previous studies, NfL has shown potential as a reliable biomarker, not only with regard to diagnostics but also prognostics and monitoring in ALS.^10^, ^11^ Yet, since NfL is elevated in many conditions, the interpretation of NfL should always be evaluated with caution and with respect to the clinical context.^12^, ^13^

Transcranial magnetic stimulation (TMS) is another approach that has gained acceptance in ALS as a method to assess upper motor neuron (UMN) involvement. The paired‐pulse techniques can assess cortical hyperexcitability, and especially, the short‐interval intracortical inhibition (SICI) parameter has proven promising as a potential diagnostic biomarker.^14^ A recent study showed a high sensitivity of the parallel tracking method (T‐SICIp) in early diagnosis of ALS, particularly in patients with few UMN signs.^15^

In this study, we aimed to compare the diagnostic performance of T‐SICIp and NfL measured in cerebrospinal fluid (CSF) with regard to sensitivity and specificity in patients referred with the suspicion of ALS, after a diagnosis was established at clinical follow‐up. Furthermore, we compared how T‐SICIp and NfL correlate with clinical scores.

## Methods

### Patient demographics

**Figure 1:**
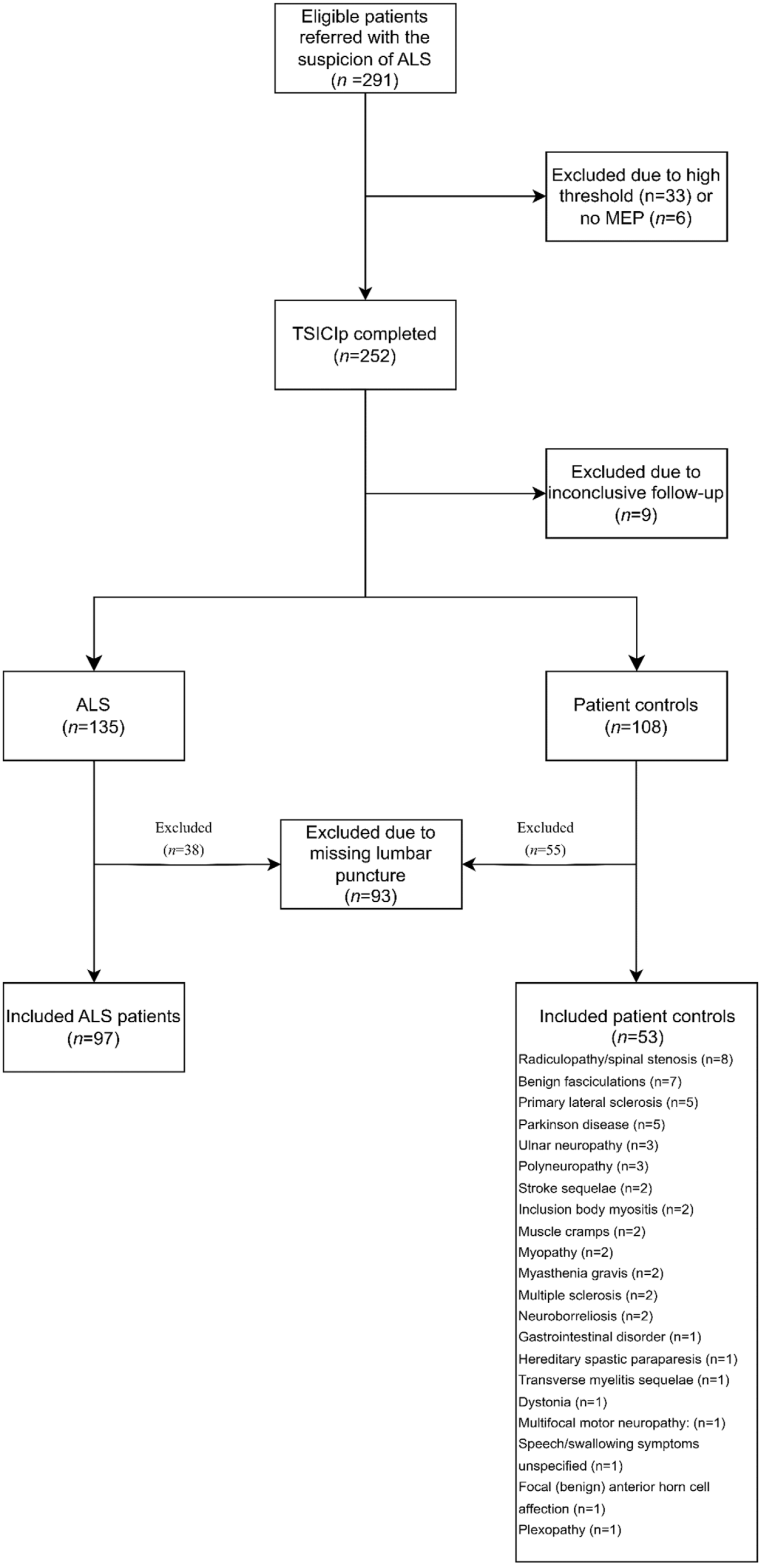
Flow chart of patient inclusion.

Two hundred ninety‐one patients referred with the suspicion of ALS were recruited between April 2018 and April 2023 from the Department of Clinical Neurophysiology at Aarhus University Hospital. Of these, 39 patients were excluded because T‐SICIp could not be recorded, either due to a high threshold or a missing motor evoked potential (MEP). ALS patients were categorized according to the Awaji criteria,^16^ later formalized as the Gold Coast Criteria at the time of inclusion, and the diagnoses for all patients had to be confirmed at clinical follow‐up. Nine patients were excluded because of inconclusive follow‐up. One hundred thirty‐five patients fulfilled the criteria for ALS, and 108 patients did not fulfill the criteria for ALS and formed the patient control group with ALS‐mimicking disorders. Of these 93 patients (38 ALS patients and 55 patient controls) were excluded due to missing lumbar puncture. The final cohort comprised 150 patients which included 97 ALS patients and 53 patient controls. Figure 1 shows a flow chart with the patient inclusion. Forty‐one of the included ALS patients and 11 of the patient controls were included in two previous studies.^14^, ^15^ At the time of inclusion, the patients had not yet received a diagnosis, and therefore, they had not received treatment with Riluzole. Mean age for the patients was 67.1 (31–87) and for the patient controls 62.4 (30–84) years. Mean disease duration was 11.7 ± 7.7 months for the patients and 18.3 ± 20.8 for the patient controls. Further patient demographics are summarized in Table 1. Exclusion criteria were as follows: (1) Former central or peripheral nervous system disease and (2) contraindications to TMS or lumbar puncture. All participants signed an informed consent before entering the study, and the study was carried out in accordance with the Declaration of Helsinki II. The project was approved by the Regional Scientific Ethical Committee and the Danish Data Protection Agency. The time between the examinations described below did not exceed 1 month for each of the patients.

**Table 1:** Patient demographics.

	Patients (*n* = 97)	Patient controls (*n* = 53)	*p* values
Age, years; median (range)	69 (31–87)	65 (30–84)	0.0248*
Sex, *n* (%)	Male: 61 (62.8%) Female: 36 (37.2%)	Male: 37 (69.8%) Female:16 (30.2%)	0.940
Onset, *n* (%)	Bulbar: 42 (43.3%) Spinal: 55 (56.7%)	Bulbar: 12 (22.6%) Spinal: 41 (77.4%)	0.0368*
Disease duration, months; median (IQR)	10 (8–12)	12 (6.75–21.8)	0.217
ALSFRS‐R; median (IQR)	41 (38–44)	45 (41–48)	1.18 × 10^−5^*
Upper motor neuron score; median (IQR)	6 (1–9)	0 (0–7)	0.00373*
MRC score; median (IQR)	73.5 (67–77)	78 (74–80)	3.66 × 10^−5^*

Statistically significant *p* values <0.05 are marked with *.

### Clinical scores

Patients were evaluated with the revised ALS functional Rating Score (ALSFRS‐R),^17^ and a detailed neurological examination was performed. Muscle strength was evaluated by the Medical Research Council (MRC) score for shoulder abduction, elbow flexion, wrist extension, index finger abduction, thumb abduction, hip flexion, knee extension, and ankle dorsiflexion yielding a maximum total score of 80. UMN involvement was evaluated by a modified Penn UMN score (UMNS).^18^ Points were given for an abnormal jaw‐jerk reflex, palmomental sign, pseudobulbar affect, increased deep tendon reflexes (biceps, triceps, patellar and ankle), pathological reflexes (Hoffman's and Babinski's signs), clonus, and spasticity. The maximum score was 27.

### Transcranial magnetic stimulation (TMS)

Patients were seated in an armchair and instructed to remain as relaxed as possible during the examination. TMS stimulation was applied focally through a figure‐of‐eight shaped magnetic coil connected to two Magstim 200 magnetic stimulators via a BiStim module (Magstim, UK). The coil was placed on the head over the motor cortex, at an angle of approximately 45° to the sagittal plane. MEPs were recorded using surface electrodes placed over the first dorsal interosseous (FDI) muscle in a belly‐tendon configuration on the side opposite to the stimulation site. The optimal coil placement was determined by recording MEPs while moving the coil position until the highest peak‐to‐peak amplitude of the MEP (hot spot) was reached, and this was marked with a semi‐permanent pen to ensure accurate coil positioning throughout the testing. The MEP responses were recorded and tracked using the QtracS component of the QtracW software (©UCL, distributed by Digitimer Ltd.) using QTMSG‐12 recording protocols (QTMS Science).

### Threshold‐tracking short‐interval intracortical inhibition

Resting motor thresholds (RMTs) for a 200 μV (RMT200) peak‐to‐peak response were detected using a 4 → 2 → 1 tracking rule, as previously described.^19^ RMTs and the thresholds, both conditioned and unconditioned, were estimated from the stimuli and responses by weighted logarithmic regression.^19^, ^20^ The parallel threshold‐tracking method abbreviated T‐SICIp, which has been described previously, was used,^19^ in which SICI at different inter‐stimulus intervals (ISIs) were tracked in parallel. The conditioning stimulus amplitude was set to 70% of RMT200, and the test stimuli tracked the 200 μV target. The test‐alone stimuli were delivered after each of three conditioning + test combinations, with the ISIs (1, 1.5, 2, 2.5, 3, 3.5, 4, 5, and 7 ms) in a pseudorandom order, and each of the nine paired stimuli was delivered 10 times. Data analysis as follows below is based on T‐SICIp at an ISI of 2.5 ms, since this is the ISI where maximal inhibition occurs and is proposed to be mediated by GABA_A_ receptors.^20^

### Neurofilament light chains (NfL)

As part of the patients' routine diagnostic workup, NfL was measured in CSF (500 μl) collected by lumbar puncture. The CSF was centrifuged for 10 min at 2000 × *g* before storage at −20°C. The samples underwent three freeze–thaw cycles prior to analysis and NfL concentrations were measured using a single‐molecule array (SIMOA) assay.^21^, ^22^

### Nerve conduction studies (NCS) and electromyography (EMG)

Conventional NCS and EMG were performed according to previously described methods.^23^ For NCS, the compound muscle action potential (CMAP) amplitude (peak‐to‐peak) was recorded from the first dorsal interosseus muscle (FDI). For EMG, fibrillation potentials, positive sharp waves, and fasciculations at 10 different sites were measured,^24^ and 20 motor unit potentials (MUPs) were recruited for quantitative analysis in which MUP duration and amplitude were measured, and the presence of polyphasia was noted. EMG data were only available for 72 of the ALS patients and 40 of the patient controls and NCS for 84 of the ALS patients and 41 of the patient controls.

### Statistical analysis

All analyses were done with the QtracP software and STATA 17.0. Parametric data are represented as the mean ± standard deviation and nonparametric data as the median and interquartile range. The Student *t*‐test and the Mann–Whitney U test were used to determine the significant differences between two groups for normally distributed or non‐normally distributed data, respectively. Pearson's and Spearman's correlation tests were performed for parametric and nonparametric data, respectively. The area under the curve (AUC), optimal cutoff levels, specificity, and sensitivity were calculated using receiver operating characteristic (ROC) curves. Results with *p* values <0.05 were considered significant.

## Results

### T‐SICIp and NfL in patients and patient controls

**Figure 2:**
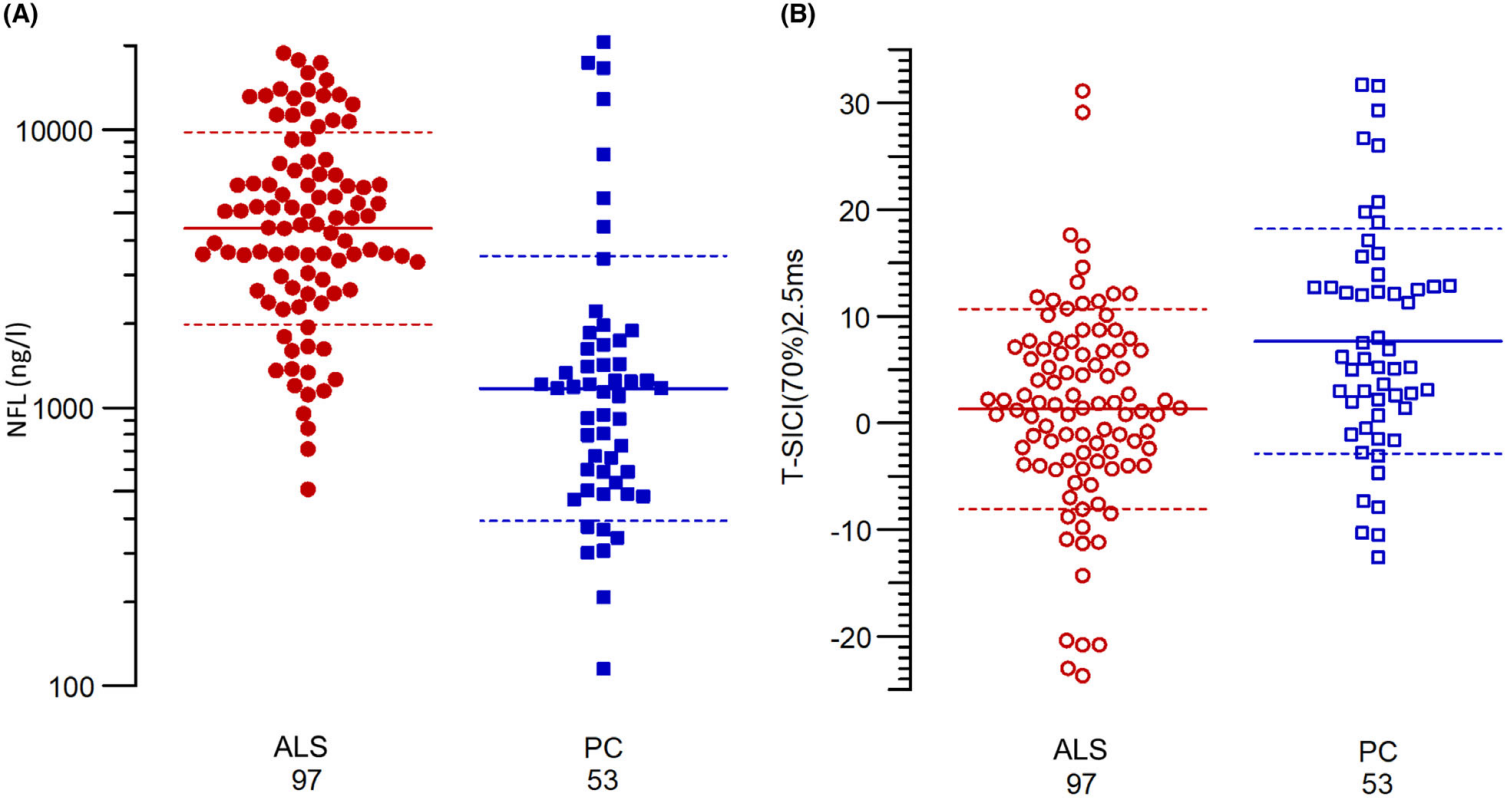
Dot plots for (A): NfL and (B): T‐SICIp at 2.5 ms. ALS patients (*n* = 97) are shown in red circles and patient controls (*n* = 53) in blue squares, NfL in filled symbols and T‐SICIp in empty ones.

Figure 2 shows dot plots for both T‐SICIp and NfL in ALS patients and patient controls. NfL was significantly higher in ALS patients (*p* = 4.4 × 10^−12^), and T‐SICIp was significantly reduced (*p* = 0.00029) compared with patient controls.

### Correlations analyses

**Figure 3:**
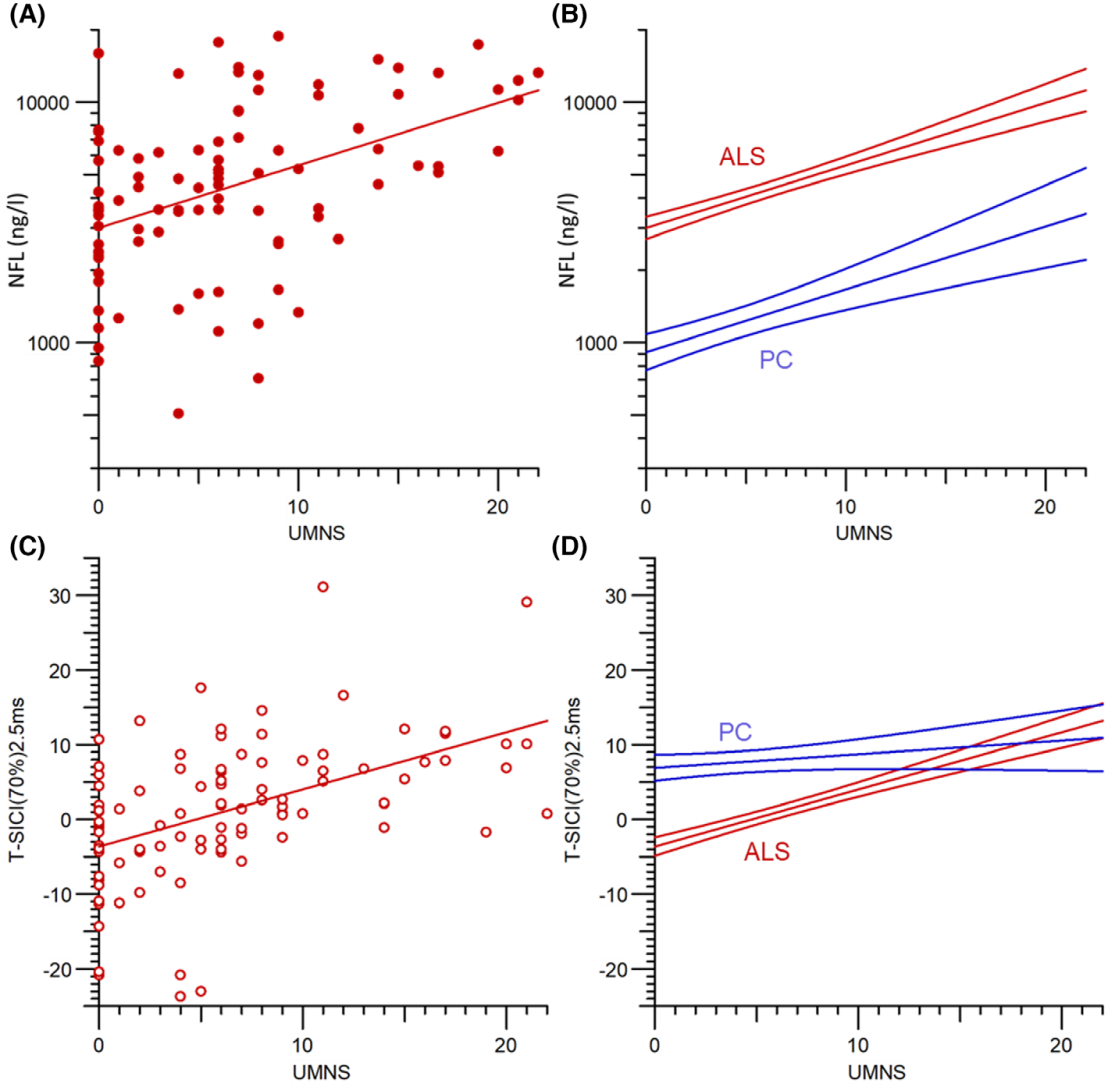
Relationships between NfL and UMNS (A and B) and T‐SICIp at 2.5 ms and UMNS (C and D). (A and C) Show individual ALS patients, while the lines show regression of NfL and T‐SICIp on UMNS. (B and D) Show regression lines ±SE for ALS patients (red) and patient controls (blue).

When considering ALS patients, both T‐SICIp and NfL correlated with UMNS (*Spearman's rho*: 0.53 [*p* = 1.9 × 10^−7^]; 0.45, [*p* = 8.2 × 10^−6^], respectively). Whereas NfL increased further to more abnormal levels with increasing UMNS score, the reduced T‐SICIp on the other hand paradoxically increased to more normal values as shown in Figure 3A,C. As opposed to NfL, T‐SICIp only discriminated between patients with low UMNS as shown in Figure 3B,D where regression lines of NfL and T‐SICIp on UMNS for both ALS patients and patient controls are plotted. NfL and T‐SICIp were uncorrelated, and neither correlated with ALSFRS‐R or MRC score. There was a weak correlation between NfL and CMAP amplitude (*rho* = −0.24, *p* = 0.029) and between NfL and fibrillations/positive sharp waves (*rho* = 0.34, *p* = 0.0033). There were no correlations between T‐SICIp and EMG parameters. Table 2 shows correlations between NfL, T‐SICIp, UMNS, ALSFRS‐R, and MRC for 97 ALS patients and EMG parameters for 72 ALS patients.

**Table 2:** Correlations expressed as Spearman Rho or Pearson correlation coefficient between NfL, T‐SICIp, UMNS, ALSFRSR, MRC (*n* = 97), and EMG parameters (*n* = 72) for ALS patients.

	NfL		T‐SICIp 2.5 ms	
	*Rho*	*p* values	*Rho* (R)	*p* values
NfL			0.049	0.64
T‐SICIp 2.5 ms	0.049	0.64		
UMNS	0.45	8.22 × 10^−6^***	0.53	1.94 × 10^−7^***
ALSFRS‐R	−0.058	0.58	−0.085	0.41
MRC	−0.093	0.37	−0.18	0.075
CMAP amplitude	−0.24	0.029**	(−0.022)	0.82
Fibrillations/positive sharp waves	0.34	0.0033**	0.067	0.58
Fasciculations	0.06	0.62	−0.14	0.24
Polyphasia	0.07	0.56	−0.071	0.56
MUP amplitude	0.009	0.90	0.006	0.92
MUP duration	0.079	0.51	(0.075)	0.54

Pearson's R indicated in parentheses. Statistically significant correlations are shown as: ***p < 0.001 and **p < 0.01.

### Discrimination of ALS patients from patient controls

**Figure 4:**
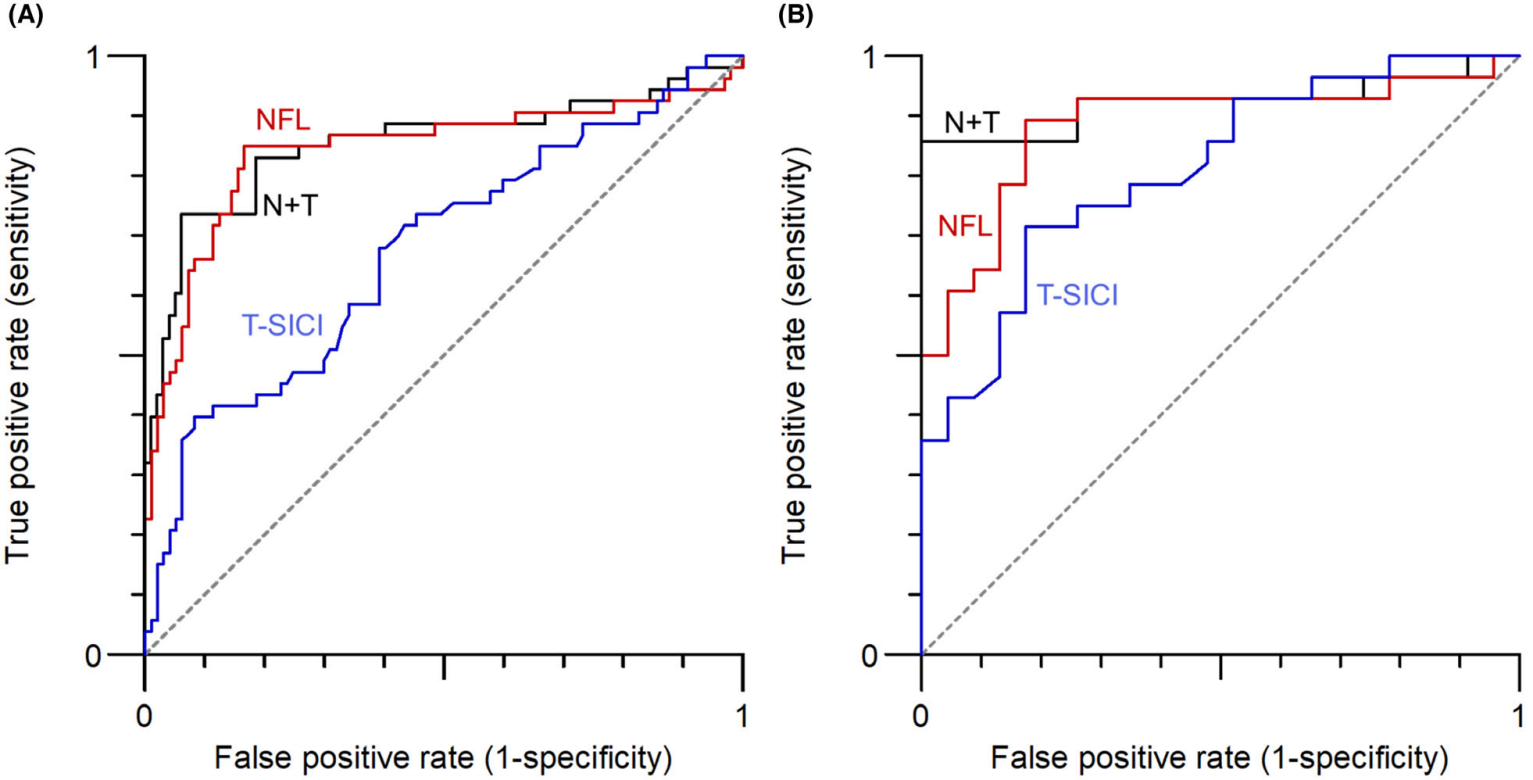
ROC curves for (A): All patients (97 ALS patients and 53 patient controls) and (B): Patients with UMNS = 0 (23 ALS patients and 28 patient controls). NfL is shown in red and T‐SICIp in blue. The black lines show the ROC curve for a combination of NfL and T‐SICIp, that is, T‐SICIp‐50 × Log_10_(NfL).

**Figure 5:**
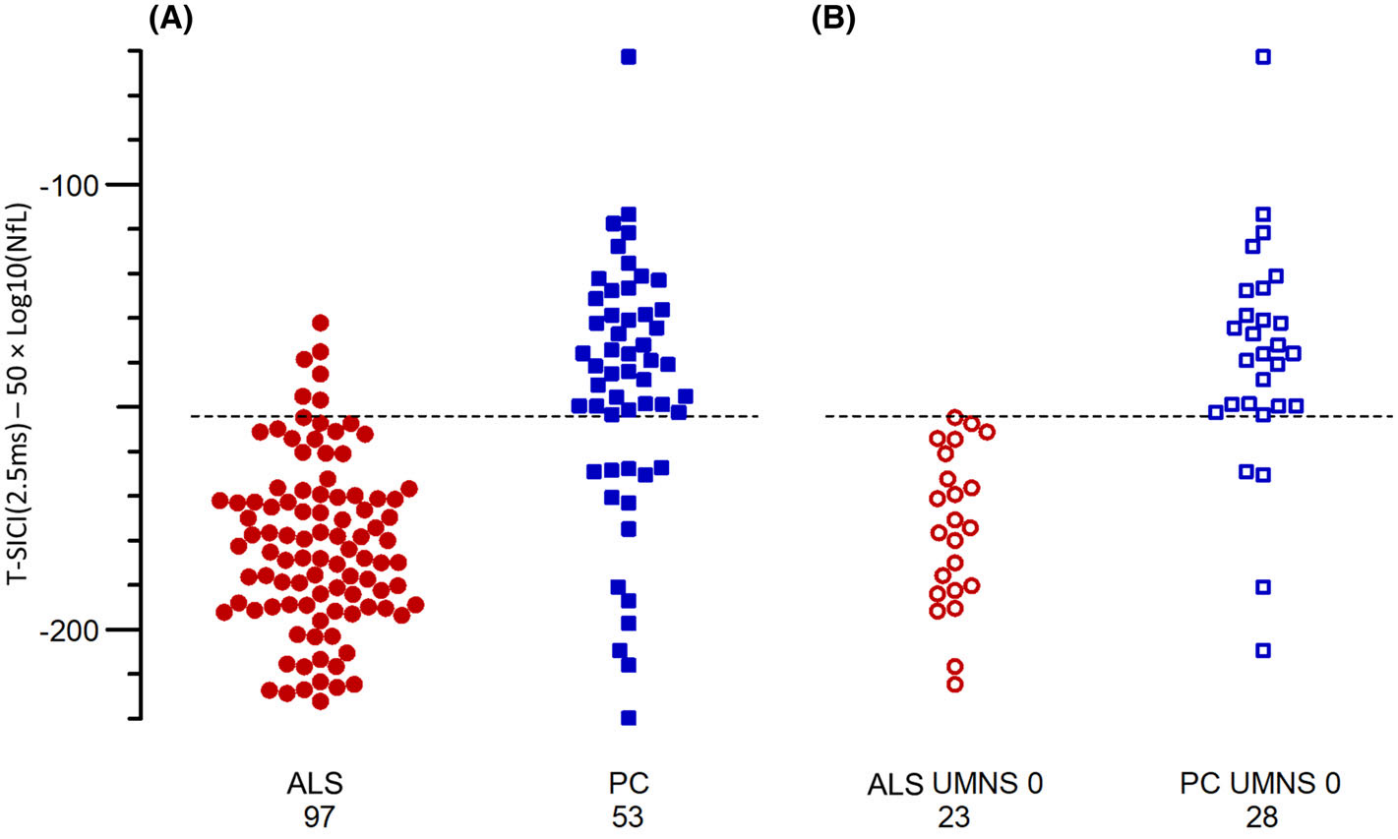
Dot plots for the combination of NfL and T‐SICIp for ALS patients and patient controls. The cutoff for optimum sensitivity and specificity is illustrated with a dashed line. ALS patients are shown in red circles and patient controls in blue squares. All patients (97 ALS patients and 53 patient controls) are shown in filled symbols and patients with UMNS = 0 (23 ALS patients and 28 patient controls) in empty ones.

Figure 4A,B shows ROC curves for NfL and T‐SICIp for all patients and for patients with an UMNS of 0, respectively. Tables 3 and 4 show the corresponding AUC, sensitivities, and specificities. As a single parameter, NfL yielded the highest AUC and corresponding sensitivity and specificity both when considering all ALS patients vs. patient controls (0.842, 84.9%, 83.5%, respectively) and when considering patients with a UMNS of 0 (0.884, 89.3%, 82.6%). T‐SICIp yielded a lower AUC of 0.675 when considering all patients and 0.811 when considering patients with a UMNS of 0. When combining NfL and T‐SICIp, higher AUCs of 0.854 and 0.922 and importantly specificities of 93.8 and 100 were found when considering all patients and patients with no UMN signs, respectively. NfL and T‐SICIp values were combined, using the expression T‐SICIp – 50 × Log_10_(NfL). The factor of 50 was found to give the highest AUC both for all patients and for those with UMNS 0. The cutoff for optimum sensitivity and specificity is illustrated in Figure 5 which shows dot plots for the combination of NfL and T‐SICIp for patients and patient controls both for all patients and for patients without UMN symptoms.

**Table 3:** Area under the curve (AUC), cutoff values, sensitivity, and specificity for all (ALS patients (*n* = 97) and patient controls (*n* = 53).

	AUC	Cutoff	Sensitivity	Specificity
NfL	0.842	2239	84.9	83.5
T‐SICIp	0.675	11.9	39.6	91.8
NfL and T‐SICIp combined	0.854	−152.1	73.6	93.8

**Table 4:** Area under the curve (AUC), cutoff values, sensitivity, and specificity for ALS patients (*n* = 23) and patient controls (*n* = 28) with UMNS = 0.

	AUC	Cutoff	Sensitivity	Specificity
NfL	0.884	1768	89.3	82.6
T‐SICIp	0.811	2.25	71.4	82.6
NfL and T‐SICIp combined	0.922	−152.1	85.7	100

## Discussion

This is the first study to compare two promising biomarkers, NfL and TMS, in ALS. We found that there were significant differences in both NfL and T‐SICIp between ALS patients and patient controls consistent with previous studies.^14^, ^15^, ^25^, ^26^, ^27^ Both correlated with UMN involvement, but whereas NfL increased to more abnormal values with increasing UMNS, T‐SICIp increased to normalized values. We found that NfL was the most sensitive parameter in distinguishing ALS patients from patient controls. When combining NfL and T‐SICIp, we found a higher AUC and specificity than for NfL alone, in particular for patients without UMN symptoms.

### Correlations between NfL, T‐SICIp, and UMNS

The paradoxical correlation between T‐SICIp and UMNS has also been found in previous studies, where it was suggested that the most disinhibited motor neurons progressively degenerate while the inhibitory neurons survive.^14^, ^15^ The positive correlation between NfL and UMNS is in agreement with previous studies which suggested that elevation in NfL levels in patients with UMN involvement might reflect corticospinal tract degeneration.^27^, ^28^, ^29^, ^30^, ^31^ Even though both NfL and T‐SICIp correlated with UMNS, we found no correlation between NfL and T‐SICIp indicating that they reflect different types or locations of abnormalities. Whereas T‐SICIp is only influenced by UMN involvement, NfL levels are likely to rise by either lower motor neuron (LMN) or UMN lesions, supported by the positive correlation of NfL levels and signs of active denervation in the form of fibrillation potentials and positive sharp waves and the negative correlation between increasing NfL levels and decreasing CMAP amplitude (Table 2). The correlation between NfL and LMN has also been reported by previous studies,^27^, ^32^ and elevated NfL levels have been found in other neurological diseases affecting lower motor neurons such as polyneuropathies.^33^

### Diagnostic performance of NfL and T‐SICIp

When comparing NfL and T‐SICIp, we found that NfL was the most sensitive parameter in distinguishing ALS patients from patient controls, with the highest AUC (0.842) determined with ROC curves, yielding the highest sensitivity (84.9) and specificity (83.5). These results for NfL are in agreement with a meta‐analysis by Sferruzza et al,^34^ which included multiple studies that investigated blood and CSF NfL concentrations and evaluated their ability to distinguish ALS patients from ALS mimic disorders. Most of the included studies also recruited patients referred with the suspicion of ALS consecutively, and the patient control groups were similar to this study. They found that NfL levels both in blood and in CSF were consistently higher in ALS patients compared to ALS mimics. NfL in blood and CSF correlated but the diagnostic performance of NfL in CSF was higher than that in blood. For the 10 studies of CSF NfL, they found a pooled sensitivity and specificity of 0.83 and a summary AUC of 0.90, and for the five studies of blood NfL, they found a pooled sensitivity of 0.85, a pooled specificity of 0.75, and a summary AUC of 0.78. This is in contrast with another study that compared CSF NfL and blood NfL in 75 ALS patients and 60 ALS mimics, which also found a correlation between them but almost identical AUCs of 0.94 and 0.93, respectively, and concluded that they were equally suited for the differential diagnosis of ALS.^35^

The AUC of T‐SICIp was comparable to one study^15^ but lower than what was found in another similar study.^14^ A reason for this could be that only patients with a suspicion of ALS who had NfL measured in spinal fluid as part of their diagnostic workup were recruited in this study. Thus, the patient controls represent a group of patients, where the clinicians had a particularly strong suspicion of ALS, in which it was necessary to measure NfL in spinal fluid because they were difficult to differentiate clinically from ALS. If a broader group of patient controls had been included, the differentiation between patients and patient controls with T‐SICIp may have been even more distinct.

A general problem with NfL as opposed to T‐SICIp is the fact that NfL levels are likely to rise with both UMN and LMN signs which often co‐exist. So even when UMN symptoms are absent, NfL can be abnormally high. Thus, an elevated NfL level is a sign of neurodegeneration, but it does not provide evidence of where the lesion is, and NfL will never be able to confirm UMN lesions when there are only LMN lesions and vice versa. We found that the combination of NfL and T‐SICIp, yielded higher AUCs of 0.854 and 0.922 and importantly specificities of 93.8 and 100 than NfL alone when considering all patients and patients without UMN symptoms, respectively. In a clinical context, this is useful since it suggests that the combination of NfL and T‐SICIp compensates for the lower specificity found for NfL, in particular for patients with no UMN signs.

## Limitations

There are limitations in this study to address. First, there was a limited number of study participants, although comparable with other similar studies. Second, as mentioned previously, we restricted the inclusion of patients to those who had NfL measured in spinal fluid as part of their diagnostic workup. In a clinical context, it is not necessary to perform lumbar puncture on all patients suspected of ALS, especially if the suspicion is vague or if another diagnosis is established as part of their diagnostic process. Thus, the patient controls included in our study were all particularly difficult to differentiate clinically from ALS, and this could have impacted the results. This limitation could also partially explain the large proportion of patients included without upper motor neuron signs (23.7% of ALS patients and 52.8% of patient controls). Third, patients were not included at the same time interval from symptom onset but when they were referred, and this could have affected the correlations for T‐SICIp and NfL.

## Conclusion

To conclude, both NfL and T‐SICIp have limitations and are not specific for ALS, but they seem to have different properties as biomarkers that make them complement each other well in improving the diagnosis of ALS. As a single parameter, NfL was most sensitive in distinguishing ALS patients from patient controls yielding the highest AUC compared to T‐SICIp. However, when considering the combination of NfL and T‐SICIp, the diagnostic outcome measures were increased, in particular for patients with no UMN symptoms who in a clinical setting are the most challenging patients to diagnose. A clear advantage of TMS as compared to CSF NfL is that it is less invasive, and for future studies, it would be relevant to include blood NfL and compare it with TMS. Moreover, as mentioned previously, more studies show that NfL is a reliable biomarker with regard to monitoring of ALS, and in the future, it would be relevant to assess, how T‐SICIp performs as a longitudinal biomarker. Hopefully, this can also help us gain more knowledge of the pathophysiology behind ALS and ultimately bring us one step closer toward finding a cure.

## Author Contributions

ABJ, HB, and HT contributed to conception and design of the study. ABJ, HB, HP, and HT contributed to acquisition and analysis of data. All authors contributed to interpretation of the data and drafting the manuscript.

## Conflict of Interest

HB and JH receive from UCL shares of the royalties for sales of the Qtrac software used in this study. HB and MK are shareholders of QTMS Science Ltd., which licenses the QTMSG‐12 recording protocols used. The other authors report no competing interests.

## Data Availability

The data that support the findings of this study are available from the corresponding author, upon reasonable request.
